# Predicting the Ability of Preclinical Diagnosis To Improve Control of Farm-to-Farm Foot-and-Mouth Disease Transmission in Cattle

**DOI:** 10.1128/JCM.00179-17

**Published:** 2017-05-23

**Authors:** Noel Nelson, David J. Paton, Simon Gubbins, Claire Colenutt, Emma Brown, Sophia Hodgson, Jose L. Gonzales

**Affiliations:** a The Pirbright Institute, Pirbright, Woking, United Kingdom; b Met Office, Exeter, United Kingdom; c Wageningen Bioveterinary Research, Lelystad, the Netherlands; University of Tennessee

**Keywords:** FMD, surveillance, transmission, early detection, sensitivity, diagnostics, PCR, foot-and-mouth disease virus

## Abstract

Foot-and-mouth disease (FMD) can cause large disruptive epidemics in livestock. Current eradication measures rely on the rapid clinical detection and removal of infected herds. Here, we evaluated the potential for preclinical diagnosis during reactive surveillance to reduce the risk of between-farm transmission. We used data from transmission experiments in cattle where both samples from individual animals, such as blood, probang samples, and saliva and nasal swabs, and herd-level samples, such as air samples, were taken daily during the course of infection. The sensitivity of each of these sample types for the detection of infected cattle during different phases of the early infection period was quantified. The results were incorporated into a mathematical model for FMD, in a cattle herd, to evaluate the impact of the early detection and culling of an infected herd on the infectious output. The latter was expressed as the between-herd reproduction ratio, *R_h_*, where an effective surveillance approach would lead to a reduction in the *R_h_* value to <1. Applying weekly surveillance, clinical inspection alone was found to be ineffective at blocking transmission. This was in contrast to the impact of weekly random sampling (i.e., using saliva swabs) of at least 10 animals per farm or daily air sampling (housed cattle), both of which were shown to reduce the *R_h_* to <1. In conclusion, preclinical detection during outbreaks has the potential to allow earlier culling of infected herds and thereby reduce transmission and aid the control of epidemics.

## INTRODUCTION

Foot-and-mouth disease (FMD) is a contagious viral disease caused by the foot-and-mouth disease virus (FMDV). It affects domestic ruminants such as cattle, sheep, and goats as well as pigs and other cloven-hoofed wild and domestic mammals. If not brought under control, the disease can spread rapidly, resulting in significant economic impacts with regard to trade and animal productivity (1). For example, during the 2001 epidemic in the UK, in excess of 2,000 cases were confirmed, leading to several million animals being culled. Some £2.5 billion was paid by the government in compensation for slaughtered animals and the costs associated with the cleanup and safe disposal of animal carcasses (2).

For FMD-free countries, the focus on maintaining their FMD-free status centers on avoiding the introduction of the virus. However, once an outbreak has been declared, the emphasis changes to one of disease control through the culling of animals on farms known to be infected, tracing dangerous contacts, and implementing animal movement restrictions. The success of reactive control measures such as those mentioned above depends critically on the time between a farm becoming infected and the virus being detected and removed (3, 4). Currently, the detection of FMDV-infected farms relies on the identification and reporting of animals showing clinical signs. Although previous studies of FMDV in cattle have shown that most transmission occurs after the onset of clinical signs (4, 5), any delays in detection can potentially compromise the effectiveness of control measures. FMDV can be detected in secretions and excretions, such as blood, nasal fluid, saliva, esophageal-pharyngeal fluid, or exhaled air, from infected animals before they show clinical disease (4–7). This raises the possibility of detecting preclinical shedding of the virus, which could in turn enable earlier intervention in the transmission cycle, leading to a decreased likelihood of onward spread.

The objective of this study was to assess the potential for preclinical (early) detection of infected herds as a control tool to reduce the risk of transmission between herds during epidemics. We used data from transmission experiments to estimate the sensitivity (Se) of different sample matrices for the detection of FMDV-infected cattle throughout the incubation period. Sample matrices included individual samples, such as blood, esophageal-pharyngeal fluid, and saliva and nasal swabs, and group-level samples, such as exhaled air. These Se estimates were incorporated into a mathematical model to evaluate the efficacy of different surveillance methods (sample matrix, number of samples, and frequency of sampling) for the early detection of an infected herd and the subsequent reduction of the infectious output. This paper concludes by discussing the potential for specific preclinical samples to be used as surveillance tools to aid in reactive control measures during an epidemic and comments on their performance as tools for early detection. Although we focus on FMD, this approach could also be applicable to other diseases such as African swine fever and classical swine fever, where virus genomes can be detected before infected pigs become infectious (8).

## RESULTS

### Infection and shedding patterns.

**FIG 1:**
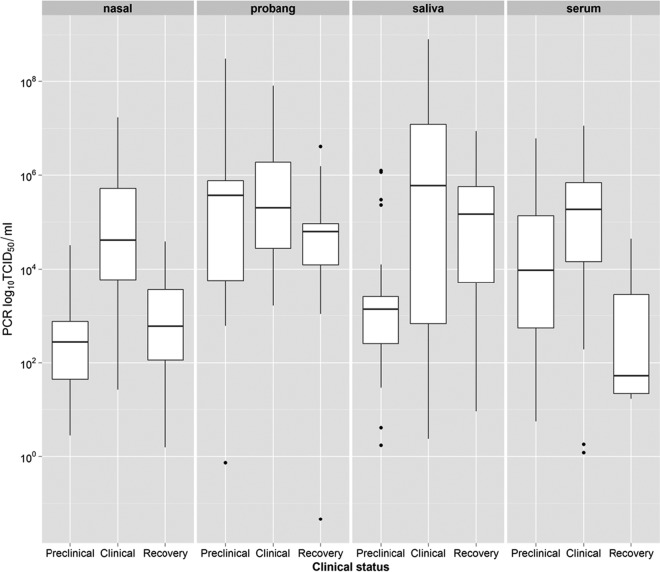
Virus shedding measured in different sample types. For analysis, the disease process is divided into preclinical, clinical, and early recovery phases. A linear mixed-regression model, where each calf identification was introduced as a random effect, was used to compare shedding levels during each phase of the disease process. When the clinical phase was used as a reference for comparison, the level of nasal shedding was significantly lower (*P* < 0.001) during both the preclinical and recovery phases. The level of shedding in saliva was lower (*P* < 0.01) in the preclinical phase, while the level of shedding in serum was lower (*P* < 0.001) in the recovery phase. No significant differences in shedding were found for probang samples (*P* = 0.06, recovery phase).

**FIG 2:**
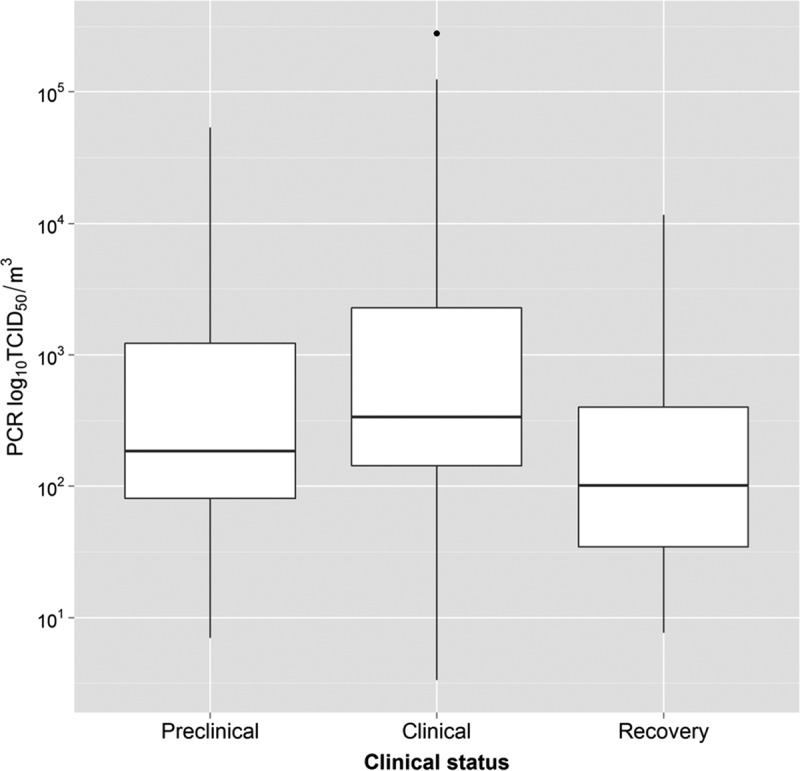
Virus shedding as measured by the cyclone air sampler. The disease process is divided into preclinical, clinical, and early recovery phases. A linear mixed-regression model, where each calf identification was introduced as a random effect, was used to compare shedding levels during each phase of the disease process. No significant differences in virus shedding were observed between the different phases. Although other air sampling devices were used, only the cyclone results were analyzed, as this sampler proved to be the most sensitive and provided more data for analysis.

In the transmission experiments, all inoculated and contact-infected calves started shedding virus (as detected by real-time quantitative reverse transcription-PCR [qPCR]) at 1 day postinoculation (dpi) or at 1 day postchallenge. With regard to inoculated calves, vesicles other than those at the inoculation sites in the tongue were observed at between 1 and 1.5 dpi, the assumed start of the clinical phase of infection. In the contact-infected calves, the clinical phase started (i.e., vesicles were observed) at between 4 and 5 dpi. Figure 1 shows the distribution of shedding measured in each of the different samples evaluated. With the exception of probang samples, the highest level of shedding was found to occur during the clinical phase. For nasal samples in particular, the level of shedding was significantly higher (*P* < 0.05) during this phase than during the preclinical or early recovery phase. Results for exhaled aerosols are shown only for the cyclone (glass) sampler because it was the most sensitive sampler (see below) and therefore provided more observations for analysis. No significant differences among the different phases (Fig. 2) were observed in this case.

### Diagnostic sensitivity of qPCR using different types of samples.

**FIG 3:**
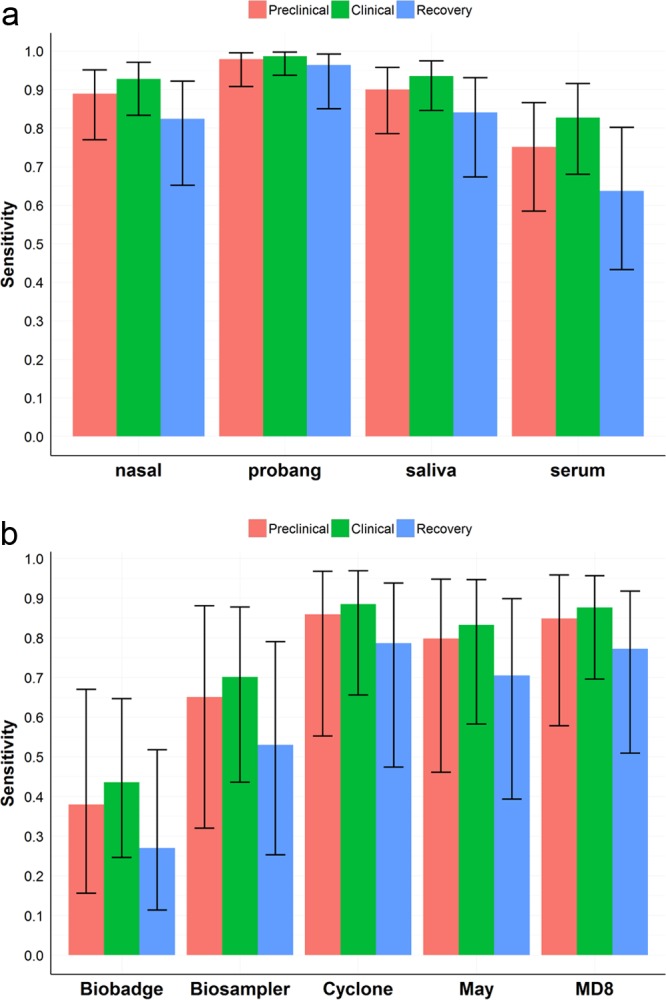
Se of different sample-qPCR combinations. Estimates of Se are stratified for the different phases of the disease process: preclinical, clinical, and early recovery. Se and 95% confidence intervals were modeled by using a logistic mixed-regression model (16), where each calf identification was introduced as a random effect. (a) Se estimates for each of the diagnostic samples evaluated; (b) Se estimates for the air samplers evaluated.

The numbers of diagnostic and air samples taken during the course of the experiments and subsequently used for this analysis are summarized in Table 1. The estimated Se values for each of the diagnostic samples evaluated were not significantly different during the preclinical, clinical, and early recovery phases. Of particular interest for this study, however, was the preclinical-phase results. All sample types taken during this phase, with the exception of serum, had a Se greater than 0.85 (85%). The probang samples gave the highest Se values, while serum samples gave the lowest values (*P* < 0.05) (Fig. 3a).

**TABLE 1:** Total numbers of samples tested for each different sample type and evaluated during the preclinical, clinical, and early recovery phases of the disease process

Sample type	No. (%) of samples during phase					
	Preclinical		Clinical		Early recovery	
	Total	Positive	Total	Positive	Total	Positive
Diagnostic^*a*^						
Nasal swabs	32	28 (88)	32	29 (91)	20	16 (80)
Probang	28	27 (96)	25	25 (100)	17	16 (94)
Saliva swabs	32	26 (81)	32	29 (91)	20	19 (95)
Serum	32	24 (75)	32	26 (81)	20	11 (55)
Air close to calves						
Airport-MD8	5	4 (80)	19	16 (83)	15	12 (80)
BioBadge	10	4 (40)	29	12 (41)	15	6 (40)
BioSampler	3	2 (67)	13	10 (77)	12	6 (50)
Cyclone	3	3 (100)	8	8 (100)	13	10 (77)
May	3	2 (67)	15	11 (73)	9	6 (67)
Room air						
Cyclone^*b*^	4	4 (100)	12	12 (100)	7	7 (100)

a Samples are taken from 16 calves (8 inoculated and 8 contact infected). Most inoculated calves were euthanized before the early recovery phase, hence the lower number of samples tested in this phase.

b Cyclone samplers used for room sampling were a glass cyclone sampler and the Coriolis sampler. Because all samples were positive (sensitivity for each phase of infection of 100%), the lower 95% confidence limits were estimated by using the Wilson score method. This method is suitable for small sample sizes (26).

In contrast to the diagnostic samples, there were significant differences (*P* < 0.05) in the Se of the different air samplers when used in close proximity to calves. The cyclone, AirPort-MD8, and May samplers, which had the highest air sampling rates, had the highest Se (Se of >0.7) during the preclinical phase. The BioBadge air sampler was shown to have the lowest Se (Fig. 3b).

**FIG 4:**
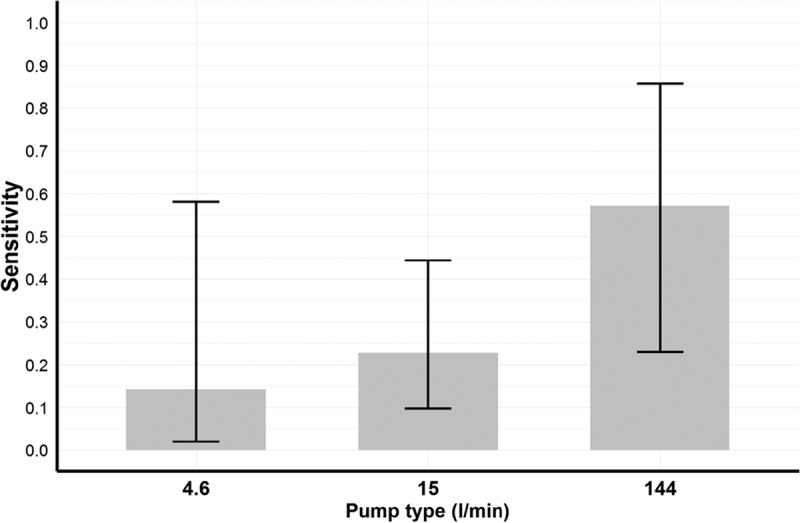
Se of filter-based air samplers with different sampling rates for preclinical detection of foot-and-mouth disease virus serotype A aerosols. Data used for these estimates were reported previously (6). Se and 95% confidence intervals were modeled by using a logistic regression model (16), which, for this data set, fit better than a mixed model. Only data for the preclinical phase were collated and analyzed.

Ambient room air was collected with the cyclone samplers, and all samples taken during the preclinical, clinical, and recovery phases were positive (Table 1). The lower 95% confidence limits (95% LCLs) for the Se at the preclinical, clinical, and early recovery phases were 0.51, 0.75, and 0.64, respectively. We also estimated the Se for room air sampling using data from a previous study (6). The highest Se was estimated for the samplers with the highest sampling rates (Fig. 4).

### Preclinical detection can be used to stop transmission between farms.

To assess the impact of preclinical detection on transmission between farms, we consider the between-herd reproduction ratio, *R_h_*. This quantity is defined as the expected number of secondarily infected farms arising from one infectious farm during its infectious period. An epidemic can sustain itself only if *R_h_* is >1, and consequently, preclinical detection will be effective at controlling spread if it is able to reduce the *R_h_* to <1.

Table 2 summarizes the expected reduction in *R_h_* as a result of active surveillance using each of the sampling matrices during epidemics. As observed during the 2001 UK epidemic, clinical inspection (based mainly on farmer reports) does not reduce the infectious output (*R_h_* = 1.54) of infected farms to a level sufficient to control spread. The implementation of preclinical surveillance using any of the evaluated sample matrices reduced the *R_h_* to <1 for sampling at a frequency of 10 animals per farm once a week. When using air samplers, daily sampling would be required to reduce the *R_h_* to <1. Similar results (i.e., *R_h_* of <1) were observed during the sensitivity analysis, when a shorter incubation period and a longer infectious period were used. However, the sample size had to be doubled in this case (Table 2). For all surveillance methods, increasing either the number of samples taken (see Fig. S1 in the supplemental material) or the frequency of sampling (Fig. S2) results in a larger reduction in *R_h_*.

**TABLE 2:** Reduction of the transmission potential of an infected herd due to implementation of preclinical detection and subsequent culling^*a*^

Scenario and type of sample used	Sampling frequency per wk	Sample size (no. of samples)^*b*^	*R_h_*	Sample size (no. of samples)^*b*^	*R_h_*
No surveillance			3.2		3.2
Baseline transmission parameters					
Reporting clinical cases	Detection delay of 8 days				1.5
Clinical inspections	Twice	10	1	20	0.5
Nasal swab	Once	5	0.8	10	0.3
Probang	Once	5	0.6	10	0.2
Saliva swab	Once	5	0.8	10	0.3
Serum	Once	5	1.1	10	0.4
Air (herd)^*d*^	Daily	0.5	1.4	1	0.6
Shorter latent period (2.25 days) and longer infectious period (4.85 days)^*c*^					
Reporting clinical cases	Detection delay of 8 days				1.7
Clinical inspections	Twice	15	0.7	20	0.5
Nasal swab	Once	10	0.5	15	0.2
Probang	Once	10	0.4	15	0.2
Saliva swab	Once	10	0.5	15	0.2
Serum	Once	10	0.8	15	0.4
Air (herd)^*b*^	Daily	1	1.2	2	0.5

a The transmission potential of a herd is expressed in terms of the reproduction ratio *R_h_*.

b Different sample sizes are shown as examples of the effect of sample size on *R_h_*.

c This means that cattle become infectious 2.25 days before showing clinical signs. This was done for the sensitivity analysis.

d The Se of air sampling was made conditional on a minimum prevalence of infected animals (incubation period) of 10%. For prevalences of <10%, Se is 0.

## DISCUSSION

This study attempted to evaluate the potential for preclinical diagnosis during reactive surveillance to bring about a reduction in the risk of between-farm transmission of FMD. Results obtained from analyses of the virus levels in diagnostic samples indicate that, with the exception of probang samples, the majority of virus shedding occurred during the clinical stage of infection. Airborne virus shedding, as measured by the cyclone air sampler, suggested that this is also true for exhaled aerosol emissions. More importantly for this paper, however, is virus shedding during the preclinical stage of infection. First, the results show that early-phase virus shedding (i.e., during the preclinical phase) can be detected by using a range of diagnostic and aerosol samples, with a high degree of sensitivity. Second, these samples can be used as part of emergency surveillance activities for the early detection of infected herds, thereby minimizing the risk of further transmission between herds.

In particular, shedding levels in nasal swabs were significantly higher during the clinical phase than during the preclinical and early recovery phases. Similarly, virus levels recovered in aerosols were also higher (although not significantly so) during the clinical phase. These differences in shedding levels in both nasal and aerosol samples might be associated with the previously reported higher probability of transmission during the clinical phase than during either the preclinical or the early recovery phase (4, 9). These observations suggest that nasal shedding could be used as a potential correlate of infectiousness in cattle. For example, the virus load in nasal samples could be readily used as a measure of infectiousness when evaluating vaccine efficacy during challenge experiments.

The use of blood samples for reactive surveillance and detection of infected herds before animals show clinical signs was demonstrated during the 2007 UK FMD epidemic (10). Our results are in agreement with those empirical findings; furthermore, we have quantified the Se of using blood samples as well as other diagnostic samples as preclinical biomarkers. These Se estimates are necessary to define the sample size and frequency of visits during reactive surveillance. Air sampling in closed environments was also evaluated for preclinical detection. For this variable, the Se appears to depend on the volumes of air sampled, with devices with higher sampling rates, such as the cyclone sampler or the sampler evaluated previously by Pacheco et al. with a sampling rate of 144 liters/min (6), showing the highest Se.

There are, however, some fundamental challenges associated with sampling in the field as opposed to sampling in a controlled experiment. For animals at pasture in the open air, the virus aerosol can be rapidly dispersed (or mixed), and sampling to capture enough virus to be detected in situations where a few animals have become infected will present a considerable challenge. Air sampling within an enclosed area such as a milking parlor is more practical and, due to slower dilution and removal of the virus aerosols, is likely to greatly increase the chances of detecting the virus. Detection sensitivity will be improved by sampling large volumes of ambient air using samplers, such as the cyclone sampler, which were previously shown to detect airborne viruses under field conditions (11, 12).

Any of the diagnostic samples evaluated could be used for the early detection of infected herds. Ideally, surveillance methods need to be quick, easy to employ, and as noninvasive as possible. These considerations rule out active clinical inspections and collection of probang and serum samples. They may also rule out the use of nasal sampling, as it can be very intrusive. In contrast, saliva and aerosol samplings are relatively noninvasive, and for aerosol samples, sampling instruments may be deployed in communal areas such as milking parlors. As well as using swabs, it might be possible to develop passive collection systems based on baits or licks attractive to cattle, as reported previously for pigs and wild boar (13). Both methods may be utilized by farmers and farm staff, which will boost surveillance resources and reduce the need for surveillance personnel to enter the farms to manipulate animals, thereby improving biosecurity. In addition, if either of these methods were to be employed in conjunction with a pen-side test used to detect virus genomes (14), rapid confirmation of infection may be obtained, speeding up the surveillance process even further.

Our results are based on experiments undertaken in controlled laboratory environments and may consequently differ in some respects from the real-world conditions of an outbreak. Although the models used in this paper are analytically robust (15, 16), the diagnostic and transmission parameters that are used to inform the model have been quantified experimentally. Bearing this in mind, a more conservative approach was adopted, using the lower confidence limits of the Se estimates for the evaluations. Nevertheless, field validation of the parameters might be necessary to improve our confidence in the model output. In addition, much of the fundamental work for this paper was undertaken using a FMDV serotype O strain, and transmission and disease characteristics may be different for other serotypes or strains (17–20). To account for the possible differences in transmission dynamics, we evaluated the surveillance strategies assuming a shorter latent period and, consequently, a longer infectious period and a higher within-herd basic reproduction ratio, *R*_0_. The results still indicated that early detection is possible. In addition, we also quantified the diagnostic Se for air sampling using data reported previously for another serotype (serotype A) (6).

The spread of FMD can be so rapid that by the time a sick animal has been identified and infection has been confirmed, several other animals or farms may already have been exposed to the virus. Therefore, the rapid identification of secondarily infected farms is essential if we are to limit the onward spread of disease. Current control measures involve the culling of animals on farms where the virus is detected (based mainly on clinical reporting), restrictions on transport on and off infected premises, and screening and tracing of dangerous contacts. If this response is not effective, additional preemptive culling or vaccination programs are employed to curb the epidemic. These policies do not, however, consider preclinical identification, and the results highlighted in this paper and the experience of the outbreak in the UK in 2007 (10) suggest that the fast identification and removal of infected animals on farms where animals do not yet show clinical signs can result in a reduction of the risk of onward transmission and possibly the need for preemptive culling. It should be noted, however, that the effectiveness of the preclinical approach highlighted in this paper remains reliant upon the frequency of surveillance visits as well as the number of animals sampled.

To conclude, we show that reactive surveillance for the preclinical detection of infected herds has the potential to be a valuable alternative control tool with which to improve our ability to interrupt the cycle of infection and transmission of FMDV during epidemics and thus bring about a speedier and efficient end to epidemics.

## MATERIALS AND METHODS

### Animal experiments.

Data for this study were obtained from a series of transmission experiments that were performed at The Pirbright Institute, Pirbright, UK, and Wageningen Bioveterinary Research (WBVR), Lelystad, the Netherlands.

### Experimental procedures.

For the first set of experiments, eight healthy conventional Holstein Friesian calves housed in the high-containment units at The Pirbright Institute were used. Four calves, housed in pairs, were inoculated by intradermolingual injection with 0.2 ml of a virus suspension containing 10^5^ 50% tissue culture infective doses (TCID_50_) of FMDV serotype O strain UKG/34/2001. At 2 dpi, inoculated calves exhibited clinical signs, and each calf was paired with two naive contact calves for a period of 24 h (contact transmission). At the end of each direct contact transmission experiment (3 dpi), the inoculated calves were euthanized, and the contact calves were moved from the room and housed individually in clean rooms for observation. Contact calves were monitored until the vesicles in their mouths started healing, at around days 6 to 8 post-contact challenge (dpc). All experimental procedures were reviewed and approved by the ethical committee at The Pirbright Institute.

For the second set of experiments, eight healthy conventional Holstein Friesian calves housed at the high-containment units at WBVR were used. Calves were housed in pairs, in independent rooms. First, four calves (two per room) were needle inoculated according to the same procedures (including the use of the same virus) performed at The Pirbright Institute. At 1 dpi, two naive contact calves were placed into each room in direct contact with two inoculated calves for a period of 48 h. At the end of the contact challenge at 3 dpi, the inoculated calves were euthanized, and the contact calves were moved to clean rooms (two per room), where they were observed for the development of clinical signs up to 8 dpc. All experimental procedures were reviewed and approved by the ethical committee at WBVR.

### Sampling and diagnostics.

All calves were tested for the presence of FMDV genomes or antibodies against the virus before the day of inoculation. Following inoculation or contact challenge, inoculated and contact calves were monitored twice per day for clinical signs. Nasal and saliva swabs, probang (esophageal-pharyngeal fluid) samples, and serum (blood) samples (here referred to as “diagnostic samples”) were taken daily from day 1 postinoculation to the time when these calves were euthanized. For the experiments performed at The Pirbright Institute, samples were taken twice daily.

During the experiments performed at The Pirbright Institute, air samples were taken twice daily after inoculation. Air samples were collected during a 10-min period with the room ventilation turned off and using five different sampling devices. The devices had different sampling flow rates and were as follows: the AirPort-MD8 (50 liters/min), BioBadge (10 liters/min), BioSampler (12 liters/min), May Multi-Stage Liquid Impinger (55 liters/min), and glass cyclone (570 liters/min) samplers. In addition to this, two sampling approaches were evaluated: (i) close-proximity samples taken from individual calves (sampler placed ∼10 cm from the nostril, within the path of exhaled air), and (ii) ambient room samples, with the cyclone sampler being located at the center of room, ∼1.2 m from the floor. The evaluated aerosol samplers were previously used for the detection of FMDV aerosols, and technical descriptions of these samplers were reported previously (21, 22).

Air sampling at WBVR was performed by using the AirPort-MD8 device for the close-proximity samples and a commercially available cyclone sampler (Coriolis) with a sampling flow rate of 300 liters/min for the ambient room samples. The cyclone sampler was placed close to the rooms' air exhaust, ∼1.7 m from the floor. Sampling was performed for 10 min, with the room ventilation turned on.

Both clinical and air samples were tested for the presence of virus genomes by using qPCR. For virus load quantification, 10-fold dilutions of the virus supernatant with a known titer (TCID_50_), determined by virus titration in bovine thyroid cells, were prepared as standard curves. qPCR results were expressed as the equivalent TCID_50_ per milliliter (23) for diagnostic samples or the equivalent TCID_50_ per cubic meter of air for air samples.

### Additional air sampling data.

Data reported previously by Pacheco et al. (6) on the use of air sampling in experimental rooms to assess the diagnostic potential of air sampling for the detection of FMDV were also used for analysis in this study. Those authors described the use of air samplers for the preclinical detection of FMDV in experimental rooms housing calves infected with FMDV serotype A. One calf was housed per room, and samplers with different sampling rates (4.6, 15, and 144 liters/min) were used to sample air from these rooms daily. Data were obtained from a total of 12 independent trials (rooms), providing a total of 36 room samples.

### Data analysis.

We first evaluated the level of shedding and quantified the diagnostic Se of qPCR using each of the different samples taken during the experiments. Next, the Se estimates were used to inform a mathematical model used to evaluate the effect of preclinical surveillance on blocking between-herd transmission.

### Diagnostic performance.

To quantify the Se of qPCR, the infection process was subdivided into three phases: (i) the preclinical phase, the period in days from challenge to the onset of clinical signs; (ii) the clinical phase, the period from the onset of clinical signs (which was defined as the appearance of one or more vesicles on the feet, mouth, tongue, or nose) to the rupture of the first vesicles; and (iii) the early-recovery phase, which was the period from the rupture of vesicles to the end of monitoring (at ∼7 or 8 dpc).

Statistical analysis was undertaken by using generalized linear mixed models (GLMMs), where each calf was included as a random effect (to account for repeated observations of the same animals). To compare shedding levels during each of the three phases of the infection process, a GLMM with a Gaussian distribution was formulated, where the relative titer in the sample as measured by qPCR was the response variable and the phase of infection was the predictor variable. For the quantification the diagnostic Se of qPCR, each sample result was classified as positive/negative, and a GLMM with a binomial error distribution was fitted (16), where the sample result (i.e., positive or negative) was the response variable. GLMMs were fitted by using the lme4 package (24) with R statistical software (25).

All the ambient air samples taken by using the cyclone sampler were positive, and consequently, it was not possible to estimate the Se for this sample-test combination using a GLMM. Instead, the lower 95% confidence limits of the Se were estimated by using the Wilson score method (26).

### Early detection of infected herds.

To evaluate the impact of using preclinical diagnosis for the early detection of infected herds during an epidemic, we combined the estimates of the diagnostic Se of the different sample type-PCR combinations in an early detection model developed previously (15). In brief, this model takes into account the infection and disease dynamics within an infected herd, where the daily prevalence of susceptible cattle, latently infected cattle, cattle in the incubation period, and infectious and recovered cattle is modeled by using a susceptible-exposed-infectious-recovered (SEIR) model, which also included a compartment for infected animals in the incubation period (see Fig. S3 in the supplemental material). This model also takes into account surveillance characteristics such as the diagnostic Se of the tests, the number of animals sampled at random (sample size) from the assessed herd, and the sampling frequency (sampling interval). All these characteristics, together with the prevalence at the time of sampling, define the Se of surveillance. The higher the Se, the lower the probability of an infected farm escaping detection within a sampling interval. Emergency surveillance starts when one (the first) infected farm is detected and control measures start. In this model, it is assumed that the spread of infection (or introduction) to at-risk farms (neighbors or dangerous contacts) takes place at any random (unknown) moment in time between the last time (a random day) when the farm was tested and the next one. A maximum of 7 days was used as a sampling interval (Table 3).

**TABLE 3:** Parameters in the mathematical model used to evaluate the effect of preclinical detection on the reduction of the risk of between-farm transmission^*a*^

Parameter	Value	Description	Reference
Within-herd infection dynamics			
Transmission rate (day^−1^)	10.15		See the supplemental material
Latent period (days)	4.65 (2.31)^*a*^		
Incubation period (days)	4.33		
Infectious period (days)	2.97 (5.94)^*a*^		
Diagnostic and surveillance parameters			
Se for nasal swabs	0.77	95% LCL of estimated Se^*b*^	This paper
Se for probang	0.91		
Se for saliva	0.79		
Se for serum	0.58		
Se for clinical inspection (days)	8	Avg time to detection during the 2001 epidemic	29
Se for clinical inspection	0.40	95% UCL of the Se^*c*^; this parameter value was used as an alternative to the 8-day delay used above	15
Se for cyclone (room air sampling)	0.55	95% LCL of estimated Se^*b*^; this Se is conditional on a minimum prevalence of infected animals (incubation period) of 10%; for a prevalence of <10%, Se = 0	This paper
No. of samples	10	No. of randomly sampled cows per herd; a minimum of 5 samples per herd was evaluated	
Frequency of sampling (no. of visits/wk)	≥1	Interval between visits to one herd	
Delay from detection to culling (days)	1	A 1-day delay between detection and culling of an infected herd was used for estimation of the reduction of infectious output	
Between-herd transmission			
*R_h_*	3.2	95% UCL of the between-herd reproduction no. (*R_h_*) estimated during the initial phase of the epidemic in 2001 in the UK	30

a Values in parentheses are parameter values changed for the sensitivity analysis and are similar to those used by Backer et al. (28).

b See Fig. 3. LCL, lower confidence limit.

c The Se of clinical inspection in partially immune populations was reported to be 0.31 (95% confidence interval, 0.22 to 0.40) (15). The upper confidence limit (UCL) of this estimate was used as an approximation to the Se in nonvaccinated populations.

These infection and surveillance parameters are combined to calculate the reduction in the infectious output due to surveillance and culling of infected animals on farms where the virus is detected and, hence, the reduction in the *R_h_*. The list of parameters and parameter values used are presented in Table 3. To be conservative and sensitive to the potential limitations of using experimental data for the estimation of the Se in the field, we used the lower 95% confidence limits of the Se estimates (Table 3) for modeling.

For sensitivity analysis, parameters determining the infection dynamics in the model, specifically the durations of the latent and infectious periods, were also changed to evaluate the effect of a longer infectious period, before the onset of clinical signs (Table 3). The model was coded by using the deSolve package (27) with R statistical software (25).

## Supplementary Material

Supplemental material
