# Supplementary material for: Predicting the Ability of Preclinical Diagnosis To Improve Control of Farm-to-Farm Foot-and-Mouth Disease Transmission in Cattle
Source: J Clin Microbiol. 2017 May 23;55(6):1671–81. doi: 10.1128/JCM.00179-17 (PMC5442523; doi:10.1128/JCM.00179-17)
Supplement: Supplemental material [file supp_55_6_1671__index.html]

Predicting the Ability of Preclinical Diagnosis To Improve Control of Farm-to-Farm Foot-and-Mouth Disease Transmission in Cattle — Supplemental material 

# Predicting the Ability of Preclinical Diagnosis To Improve Control of Farm-to-Farm Foot-and-Mouth Disease Transmission in Cattle

## Supplemental material

- Supplemental file 1 -

  Text S1 (Estimating transmission parameters for foot-and-mouth disease virus); Table S1 (Summary statistics for marginal posterior densities for transmission and latent, infectious, and incubation period parameters for foot-and-mouth disease virus in cattle); and Fig. S1 (Effect of sample size on detection and reduction of herd reproduction ratio, when sampling is done once a week), S2 (Effect of sample frequency and sample size on detection and reduction of herd reproduction ratio, when air samples are taken in communal areas such as milking parlors), and S3 (Daily prevalence of latently infected cattle, sick cattle or cattle with clinical signs, and infectious cattle)

  PDF, 691K
