# Supplementary material for: Predicting the Ability of Preclinical Diagnosis To Improve Control of Farm-to-Farm Foot-and-Mouth Disease Transmission in Cattle
Source: J Clin Microbiol. 2017 May 23;55(6):1671–81. doi: 10.1128/JCM.00179-17 (PMC5442523; doi:10.1128/JCM.00179-17)
Supplement: Supplemental material [file JCM.00179-17_zjm999095496s1.pdf]

Text S1 Estimating transmission parameters for foot-and-mouth disease virus

S1.1 Data

To estimate parameters for foot-and-mouth disease virus (FMDV) in cattle we used data from a series of one-to-one transmission experiments (1). In these experiments uninfected recipient cattle were challenged by exposure (for approximately eight hours) to FMDV-infected donor animals at two, four, six and eight days post infection of the donor. For each challenge the outcome was recorded, that is whether or not transmission occurred.

S1.2 Bayesian framework

Data from the challenge experiments were used to define an indicator variable (δ_{ij}) such that δ_{ij}=0 if transmission did not occur following the *i*th challenge by infected animal *j* (where the challenge started and stopped at $\tau_{ij}^{(0)}$ and $\tau_{ij}^{(1)}$ days post infection, respectively) and δ_{ij}=1 if it did. The probability of transmission following the *i*th challenge by infected animal *j* (i.e. δ_{ij}=1) is given by,

$$p_{ij} = 1 - \exp\left(-\int_{\tau_{ij}^{(0)}}^{\tau_{ij}^{(1)}} \beta_j(\tau)\,\mathrm{d}\tau \right). \qquad (1)$$

Here $\beta_j(\tau)$ is the infectiousness of animal *j* at τ days post infection, which is given by,

$$\beta_j(\tau) = \begin{cases} 0 & E_j \le \tau, \\ \beta_0 & E_j < \tau \le E_j + I_j, \\ 0 & \tau > E_j + I_j, \end{cases} \qquad (2)$$

where β_0 is the transmission parameter and E_j and I_j are the latent and infectious periods for the animal. The latent and incubation periods were assumed to follow a bivariate log normal distribution, that is,

$$(\log E_j, \log C_j) \sim \mathrm{MVN}(\boldsymbol{\mu}, \boldsymbol{\Sigma})$$

where,

$$\boldsymbol{\mu} = (\mu_E, \mu_C), \quad \boldsymbol{\Sigma} = \begin{pmatrix} \sigma_E^2 & \rho_{EC}\sigma_E\sigma_C \\ \rho_{EC}\sigma_E\sigma_C & \sigma_C^2 \end{pmatrix},$$

are the mean and covariance matrix (on the log scale), respectively. The infectious period was assumed to follow a log normal distribution, $g(I)$, with parameters μ_I and σ_I.

The likelihood for the challenge data (comprising the challenge outcomes, δ_{ij}, and the times at which clinical signs were first observed for each animal, C_j) can be written as,

$$L(\boldsymbol{\varphi}, \mathbf{E}, \mathbf{I}) = \prod_j \left\{ \prod_i p_{ij}^{\delta_{ij}} (1 - p_{ij})^{1-\delta_{ij}} \right\} \left\{ \int_{C_j - 1}^{C_j} f(E_j, c)\, \mathrm{d}c \right\} g(I_j), \qquad (3)$$

where $\boldsymbol{\varphi}$ is a vector of model parameters and $\mathbf{E}=\{E_j\}$ and $\mathbf{I}=\{I_j\}$ are the latent and infectious periods for each animal, respectively. Because the latent and infectious periods are not directly observed, they were included in the analysis as parameters to be estimated.

The priors for the latent, infectious and incubation period parameters were assumed to follow normal (the μs) or gamma (the σs) distributions such that the expected values for the priors yielded log normal distributions with the same mean and variance as the distributions for the latent, infectious and incubation periods in cattle presented in a meta-analysis of these parameters for FMDV serotype O (2) (see their table III). Specifically, parameters for each prior were: μ_E (mean 1.13 and shape parameter 5); σ_E (mean 0.54 and shape parameter 3); μ_I (mean 1.37 and shape parameter 5); σ_I (mean 0.49 and shape parameter 3); μ_C (mean 1.65 and shape parameter 5); and σ_C (mean 0.47 and shape parameter 3). A Uniform(-1,1) prior was used for the correlation parameter, while an informative exponential prior (with mean 5) was used for the transmission rate (3). All priors were assumed to be independent of one another.

Samples from the joint posterior distribution were generated using an adaptive Metropolis algorithm (4), modified so that the scaling factor was tuned during burn-in to ensure an acceptance rate of between 20% and 40% for more efficient sampling of the target distribution (5). Two chains of 600,000 iterations were run, with the first 100,000 iterations discarded to allow for burn-in of the chain. The chains were then thinned (taking every one hundredth sample) to reduce autocorrelation amongst the samples. Convergence was assessed visually and by examining the Gelman-Rubin statistic using the coda package (6) in R (7).

S1.3 Results

Summary statistics for the marginal posterior densities are presented in Table S1.

TABLE S1 Summary statistics for the marginal posterior densities for the transmission and latent, infectious, and incubation period parameters for foot-and-mouth disease virus in cattle.

	symbol	mean	median	percentiles	
				2.5th	97.5th
model parameters					
latent period	μ_E	1.38	1.40	0.80	1.76
	σ_E	0.49	0.46	0.27	0.91
infectious period	μ_I	0.80	0.78	0.18	1.53
	σ_I	0.55	0.50	0.14	1.31
incubation period	μ_C	1.35	1.35	1.04	1.68
	σ_C	0.43	0.41	0.26	0.72
correlation parameter	ρ_{EC}	0.67	0.74	0.00	0.98
transmission parameter	β_0	10.15	8.78	2.21	26.46
duration (days)					
mean latent period		4.65	4.55	2.77	6.99
mean infectious period		2.97	2.56	1.49	6.74
mean incubation period		4.33	4.20	3.14	6.25

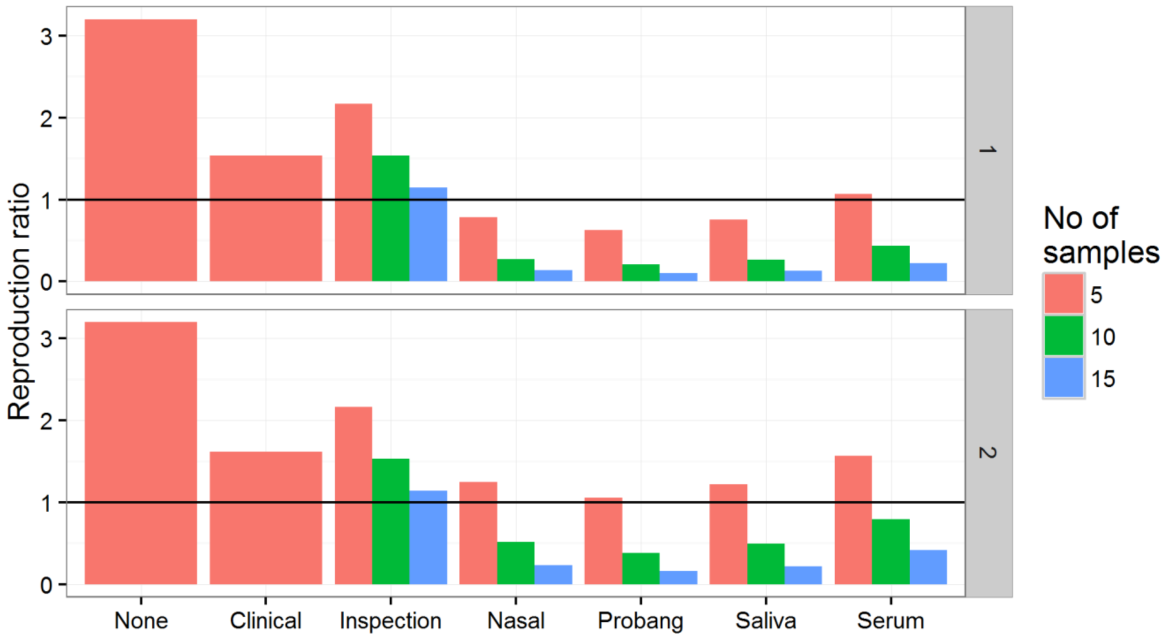

FIG S1 Effect of sample size on detection and reduction of the herd reproduction ratio (R_h), when sampling is done once a week. Panel 1 shows the results for the baseline scenario and panel 2 shows the scenario with a shorter latent period and longer infectious period (Table 1). "None" means that no surveillance is carried out and infected farm is not detected (no samples taken). "Clinical" means that farmer will notice and report clinical signs on average 8 days following the introduction of infection. "Inspection" means that active visits are done by surveillance teams where a number of animals are inspected for clinical signs of foot-and-mouth disease. Nasal, Saliva, probang and serum samples are then send to the laboratory and tested by qPCR. The horizontal line indicates the transmission threshold at R_h=1; when R_h<1, an epidemic cannot sustain itself and will die out.

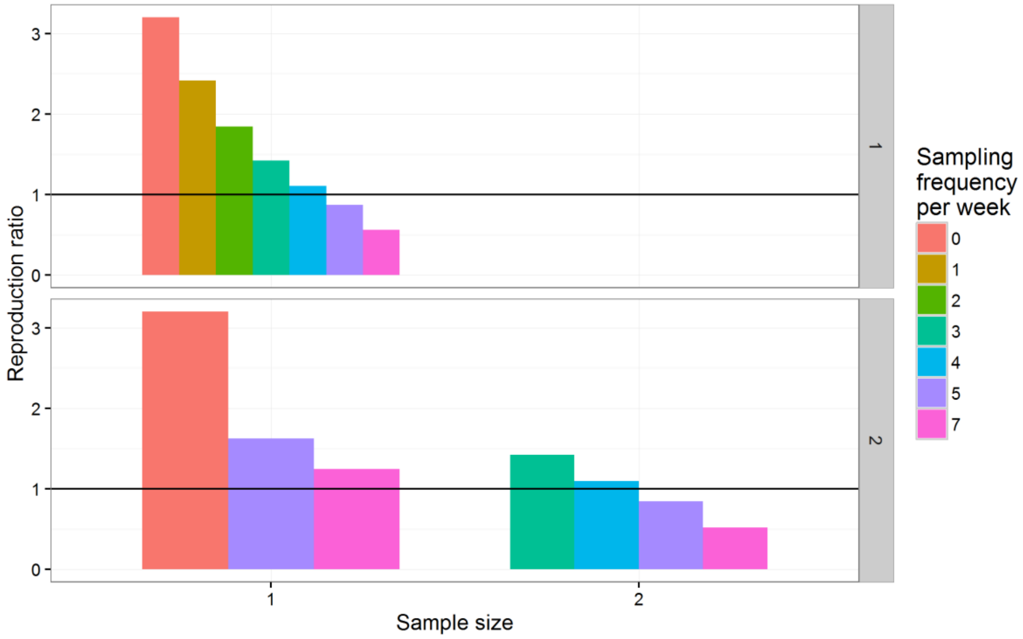

FIG S2 Effect of sample frequency and sample size on detection and reduction of the herd reproduction ratio (R_h), when taking air samples in communal areas such as milking parlours. Panel 1 shows the results for the baseline scenario and panel 2 shows the scenario with a shorter latent period and longer infectious period (Table 1). A sampling frequency of "0" means that no surveillance is carried out (i.e. no samples taken) and an infected farm will not be detected. Air samples are sent to the laboratory and tested by qPCR. The horizontal line indicates the transmission threshold at R_h=1; when R_h<1, an epidemic cannot sustain itself and will die out.

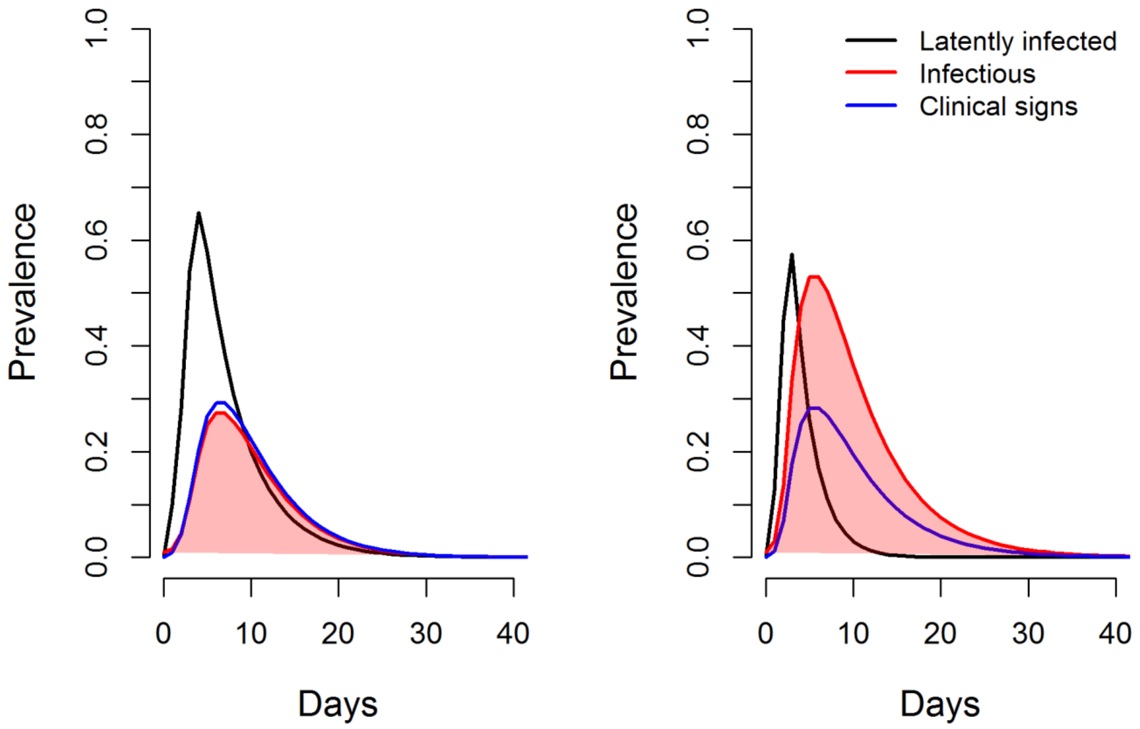

FIG S3 Daily prevalence of latently infected (black line), of sick or cattle with clinical signs (blue lines) and infectious cattle (red line). The left-hand plot shows the baseline scenario, while the right-hand plot shows the scenario with a shorter latent period and longer infectious period (see Table 1). The transmission rate and incubation period were kept the same for both scenarios. The shaded area under the red line (daily prevalence of infectious cattle) represent the overall herd infectiousness over time. The area under this curve is proportional to the between herd reproduction ratio R_h.

References

1. **Charleston B, Bankowski BM, Gubbins S, Chase-Topping ME, Schley D, Howey R, Barnett PV, Gibson D, Juleff ND, Woolhouse MEJ.** 2011. Relationship Between Clinical Signs and Transmission of an Infectious Disease and the Implications for Control. Science **332:**726-729.
2. **Mardones F, Perez A, Sanchez J, Alkhamis M, Carpenter T.** 2010. Parameterization of the duration of infection stages of serotype O foot-and-mouth disease virus: an analytical review and meta-analysis with application to simulation models. Veterinary Research **41:**45.
3. **Orsel K, Dekker A, Bouma A, Stegeman JA, de Jong MC.** 2005. Vaccination against foot and mouth disease reduces virus transmission in groups of calves. Vaccine **23:**4887-4894.
4. **Haario H, Saksman E, Tamminen J.** 2001. An Adaptive Metropolis Algorithm. Bernoulli **7:**223-242.
5. **Andrieu C, Thoms J.** 2008. A tutorial on adaptive MCMC. Statistics and Computing **18:**343-373.
6. **Plummer M, Best M, Cowles K, K V.** 2006. CODA: Convergence Diagnosis and Output Analysis for MCMC. R News **6:**7 - 11.
7. **R Core Team.** 2015. R: A language and environment for statistical computing. R Foundation for Statistical Computing, Vienna, Austria.
